# EHMTI-0067. Chronic headaches in female patients with irritable bowel syndrome: just another functional condition?

**DOI:** 10.1186/1129-2377-15-S1-D20

**Published:** 2014-09-18

**Authors:** D Georgescu, C Georgescu, LA Georgescu

**Affiliations:** 1 Internal Medicine, "Victor Babes" University of Medicine and Pharmacy, Timisoara, Romania; 2 Neurology, Academic Emergency Hospital, Timisoara, Romania; 3 General Surgery, Academic Emergency Hospital, Timisoara, Romania

## Introduction

Among comorbidities associated to irritable bowel syndrome (IBS) there are headaches including migraine attacks, frequently reported in female gender.

## Aim

Assessment of vascular involvement of migraine without aura in female patients with IBS.

## Methods

30 female patients, mean age= 47,47±8,38 years, previously diagnosed with IBS (Rome II criteria), 15 predominant constipation(IBS-C) and 15 predominant diarrhea(IBS-D), complaining of mild to moderate migraine without aura ( according to International Headache Society criteria), with no records of cigarettes smoking, hypertension, diabetes mellitus, obesity or treatment requiring dyslipidemia, undertook Duplex carotidian examination with a linear 12 MHz transductor, GE Loqic 5 Expert high resolution ultrasound device. We assessed intima-media thickness (IMT) at 1cm before right and left common carotid artery(CCA) bifurcation at the posterior wall and presence and severity of atherosclerotic plaques.

## Results

12 patients(40%) exhibited no vasular issues, IMT normal (6: IBS-D and 6: IBS-C); 18 patients (60%) showed various aspects of endotelial dysfunction: abnormal IMT in 15 patients (8: IBS-D,7: IBS-C) and small nonstenotic, stable plaques in 3 patients (1:IBS-D, 2 :IBS-C) with fibrolipidic features. There was no statistically difference between the two groups neither having vs not having vascular issues (p=0,8974), nor between abnormal IMT vs presence of atherosclerotic plaques (p=0,5549).

## Conclusions

More than half of IBS female patients complaining of migraine without aura presented features of endotelial dysfunction: either increasesd IMT or patent signs of early atherosclerotic plaques. No statistically significant differences concerning vascular issues were set between the two groups: IBS-C vs IBS-D patients.

No conflict of interest.

